# Ventricular tachycardia ablation through radiation therapy (VT-ART) consortium: Concept description of an observational multicentric trial *via* matched pair analysis

**DOI:** 10.3389/fcvm.2023.1020966

**Published:** 2023-02-27

**Authors:** Francesco Cellini, Maria Lucia Narducci, Chiara Pavone, Gianluigi Bencardino, Francesco Perna, Gaetano Pinnacchio, Silvia Chiesa, Mariangela Massaccesi, Maria Antonietta Gambacorta, Stefania Manfrida, Silvia Longo, Alice Mannocci, Giuseppe Di Gregorio, Luca Boldrini, Luca Tagliaferri, Luca Indovina, Lorenzo Placidi, Gerardina Stimato, Francesco Raffaele Spera, Roberto Scacciavillani, Filippo Crea, Vincenzo Valentini, Gemma Pelargonio

**Affiliations:** ^1^Dipartimento Universitario Diagnostica per Immagini, Radioterapia Oncologica ed Ematologia, Università Cattolica del Sacro Cuore, Rome, Italy; ^2^Dipartimento di Diagnostica per Immagini, Radioterapia Oncologica ed Ematologia, Fondazione Policlinico Universitario Agostino Gemelli IRCCS, Rome, Italy; ^3^Dipartimento di Scienze Cardiovascolari, Fondazione Policlinico Universitario Agostino Gemelli IRCCS, Rome, Italy; ^4^Faculty of Economics, Universitas Mercatorum, Rome, Italy; ^5^Istituto di Cardiologia, Universitá Cattolica del Sacro Cuore, Rome, Italy

**Keywords:** radiotherapy, ventricular tachycardia, stereotactic arrhythmia radioablation (STAR), stereotactic body radiation therapy (SBRT), radioablation, clinical trial, matched pair analysis

## Abstract

**Introduction:**

Monomorphic ventricular tachycardia (VT) is a life-threatening condition often observed in patients with structural heart disease. Ventricular tachycardia ablation through radiation therapy (VT-ART) for sustained monomorphic ventricular tachycardia seems promising, effective, and safe. VT-ART delivers focused, high-dose radiation, usually in a single fraction of 25 Gy, allowing ablation of VT by inducing myocardial scars. The procedure is fully non-invasive; therefore, it can be easily performed in patients with contraindications to invasive ablation procedures. Definitive data are lacking, and no direct comparison with standard procedures is available.

**Discussion:**

The aim of this multicenter observational study is to evaluate the efficacy and safety of VT-ART, comparing the clinical outcome of patients undergone to VT-ART to patients not having received such a procedure. The two groups will not be collected by direct, prospective accrual to avoid randomization among the innovative and traditional arm: A retrospective selection through matched pair analysis will collect patients presenting features similar to the ones undergone VT-ART within the consortium (in each center independently). Our trial will enroll patients with optimized medical therapy in whom endocardial and/or epicardial radiofrequency ablation (RFA), the gold standard for VT ablation, is either unfeasible or fails to control VT recurrence. Our primary outcome is investigating the difference in overall cardiovascular survival among the group undergoing VT-ART and the one not exposed to the innovative procedure. The secondary outcome is evaluating the difference in ventricular event-free survival after the last procedure (i.e., last RFA vs. VT-ART) between the two groups. An additional secondary aim is to evaluate the reduction in the number of VT episodes comparing the 3 months before the procedure to the ones recorded at 6 months (from the 4th to 6th month) following VT-ART and RFA, respectively. Other secondary objectives include identifying the benefits of VT-ART on cardiac function, as evaluated through an electrocardiogram, echocardiographic, biochemical variables, and on patient quality of life. We calculated the sample size (in a 2:1 ratio) upon enrolling 149 patients: 100 in the non-exposed control group and 49 in the VT-ART group. Progressively, on a multicentric basis supervised by the promoting center in the VT-ART consortium, for each VT-ART patient enrollment, a matched pair patient profile according to the predefined features will be shared with the consortium to enroll a patient that has not undergone VT-ART.

**Conclusion:**

Our trial will provide insight into the efficacy and safety of VT-ART through a matched pair analysis, *via* an observational, multicentric study of two groups of patients with or without VT-ART in the multicentric consortium (with subgroup stratification into dynamic cohorts).

## Background

Monomorphic ventricular tachycardia (VT) is a life-threatening condition often observed in patients with structural heart disease. Recurrence poses a serious threat to both patient survival and quality of life (QoL). The most common cause of recurrent monomorphic VT is the presence of an ischemic scar that induces arrhythmias through re-entry mechanisms. Endocardial and/or epicardial radiofrequency ablation (RFA) represents the gold standard for VT ablation, along with medical therapy.

Stereotactic arrhythmia radioablation (STAR) uses stereotactic body radiation therapy (SBRT) for the ablation of cardiac arrhythmias, which is technically related to any arrhythmia. The present study focuses on ventricular tachycardia ablation through radiation therapy (VT-ART), applying STAR particularly focused on sustained monomorphic ventricular tachycardia.

Stereotactic body radiation therapy using VT-ART delivers focused high-dose radiation in a single fraction of 25 Gy, allowing the ablation of VT by inducing myocardial scars. The procedure is fully non-invasive; therefore, it can be easily performed in patients with contraindications to invasive ablation procedures.

Owing to the highly experimental profile of an innovative procedure such as VT-ART, too little is known to draw definitive conclusions about its efficacy, safety, and long-term results.

## Aims

Our trial aims to assess the benefits of VT-ART for the ablation of VT in patients with optimized medical therapy in whom traditional techniques, namely, RFA, have either failed to control VT recurrence or cannot be performed.

The importance of our trial relies on the identification of new management options for patients who have not responded to traditional ablation techniques or have contraindications to invasive procedures. The efficacy of VT-ART will be indirectly compared through matched pair analysis with a population of patients not treated with VT-ART, thus avoiding setting a randomized controlled trial that is considerable technically demanding and possibly premature in this research field.

## Protocol overview

With current knowledge, it is difficult to set a randomized trial between a population undergoing VT-ART (e.g., in the compassionate setting often applied for patients proposed for such an innovative procedure) and a population not undergone to that. Randomizing a patient’s accrual to VT-ART instead of the conventional option implies that some patients would skip a conventional procedure in favor of a procedure still considerate at least still non-standard, although promising. Multiple, reliable phase I and phase II trials are not available, although case series and at least one phase I/II trial have investigated VT-ART efficacy (1).

Finally, it would be difficult to define two balanced treatment arms that are suitable for a clinical randomized comparison, since patients undergoing VT-ART would have either already received RFA or are unable to receive it, and they have already undergone other standard treatments. On the other hand, setting a single-arm trial would test less efficiently the clinical of VT-ART and could imply the adoption of VT-ART only for worse clinical case presentations.

This multicenter observational study will evaluate the efficacy and safety of VT-ART. Our trial will enroll patients with optimized medical therapy, in whom endocardial and/or epicardial RFA is either unfeasible or fails to control VT recurrence. The trial is set as observational since it indirectly comparing VT-ART with a conventional procedure (i.e., RFA).

Each patient recruited for VT-ART will be profiled according to a predefined list of characteristics that will be circulated within the centers participating to the consortium to collect other patients with a similar profile, not having undergone VT-ART, for final analysis.

The peculiar setting of our trial will avoid precluding VT-ART to patients possibly taking advantage of such a therapeutic option; moreover, it will compare VT-ART with retrospective series not having receive VT-ART anyway. Furthermore, this approach will retrieve otherwise sporadic data about single or limiter case series of each center joining the consortium. This trial is not strictly investigational but can add notable scientific information to the current scenario.

The multicenter setting of this study will allow for faster recruitment of the sample size.

Multicenter recruitment will allow for a more precise assessment of the effects of VT-ART, with the aim of demonstrating that implementing such a procedure is both feasible and safe among centers. Moreover, it will increase the chances to recruit patients with profiles matching the ones in the arm undergone VT-ART.

A standardized data collection has been defined for data regarding both VT-ART and RFA.

The trial will thus both collect prospective and retrospective data about VT-ART (following the predefined standardized data collection). Prospective data collection will be applied within the consortium for the patients referred to VT-ART after the beginning of the trial; retrospective data collection will be allowed for the VT-ART procedure delivered before the formal start of the trial if the data required by the predefined standardized data collection are available for defining the primary and secondary endpoint (see following sections). Similarly, prospective and retrospective data collection will be allowed for data about conventional treatment (i.e., RFA).

## Populations

Our aim is to enroll patients with structural heart disease, in whom the presence of a myocardial scar induces refractory monomorphic VT or ventricular fibrillation (VF), as documented by either implantable cardioverter defibrillator (ICD) appropriate shocks or anti-tachycardia pacing (ATP) at ICD interrogation. We will enroll patients with recurrent VT/VF episodes despite optimal medical therapy (i.e., class III antiarrhythmic drugs) and at least one previous attempt of RFA or patients in whom ablation is not feasible, because of contraindications to the procedure or of patient intolerance. Previous percutaneous stellate ganglion blockade is neither a requirement for inclusion criteria nor exclusive criteria that will be collected in the standardized data collection for potential subgroup analysis.

Eligibility criteria are summarized in Table 1.

**TABLE 1:** Inclusion and exclusion criteria.

Inclusion criteria	1. Patients with structural heart disease and monomorphic VT refractory to optimal medical therapy and previous RFA attempts (minimum of one attempt RFA)
	2. Patients with contraindications to conventional ablation or not suitable for any non-interventional approach, refusing any surgical ablative attempt; or patients who have already undergone RFA with arrhythmogenic focus refractory to previous ablation procedures
	3. Age >18 years
	4. Candidates not suitable for heart transplantation
	5. LVEF >20%
	6. ICD implant
	7. Signed informed consent
	8. Life expectation >1 year in absence of VT
Exclusion criteria	9. ICD interrogation demonstrating polymorphic VT
	10. Patients with INTERMACS class >4
	11. Patients with LVADs
	12. Patients with ongoing neoplastic disease
	13. Previous thoracic RT with cardiac involvement
	14. Active myocardial ischemia
	15. Cardiac revascularization <120 days
	16. NYHA IV
	17. Pregnant women

## Methods/design

Our trial will investigate the effects of administering a single fraction of external beam radiation delivered through SBRT techniques at a dose of 25 Gy on patients with recurrent episodes of VT and in whom optimal medical therapy and previous RFA attempts have failed to provide benefit from VT burden control. We will perform matched pair analysis with dynamic cohorts. We will compare two groups of patients: One that has been treated by all the standard approaches but has not undergone VT-ART and a second group that has also received VT-ART. The entire enrolled group (by multicentric recruitment) will be stratified according to some predefined characteristics and analyzed by matched pair analysis, based on the characteristics of patients treated with VT-ART. Once a patient is enrolled to undergo VT-ART within the multicentric consortium, the search for two patients with similar feature profiles not undergoing VT-ART will be shared.

Matching factors taken into account are summarized in Table 2.

**TABLE 2:** Applied matching factors.

Parameter type	Subgroup options
Etiology of cardiomyopathy*	ICM
	NICM
	ACM
Age*	
Gender*	
BMI	
LVEF*	
NYHA group*	
Ventricular arrhythmia presentations*	Sustained ventricular tachycardia incessant sustained ventricular tachycardia electrical storm
Arrhythmogenic focus anatomical intracardiac location	Based on VE exit by ECG and by the activation map of VT
Diagnostic tools used for scar definition	CT
	MR
	EAM
	ECG
TA volume*	Endocardial electroanatomic substrate mapping area/volume in all cases; epicardial mapping area/volume in the case of 3D epicardial electroanatomic substrate mapping
Heart volume	cc range
Heart-to-TA volume	cc range
Previous use of amiodaron	Y/N
Number of previous RFA attempts*	
Type of previous RFA attempts	endocardial
	epicardial
Unfeasibility to repeat RFA	Y/N
Local recurrence of TA (same area of last RFA)	Y/N
Previous percutaneous stellate ganglion blockade	Y/N

TA, target area by electroanatomical mapping; BMI, body mass index; RFA, radiofrequency ablation; LVEF, left ventricle ejecting fraction; *mandatory matching factor.

**FIGURE 1:**
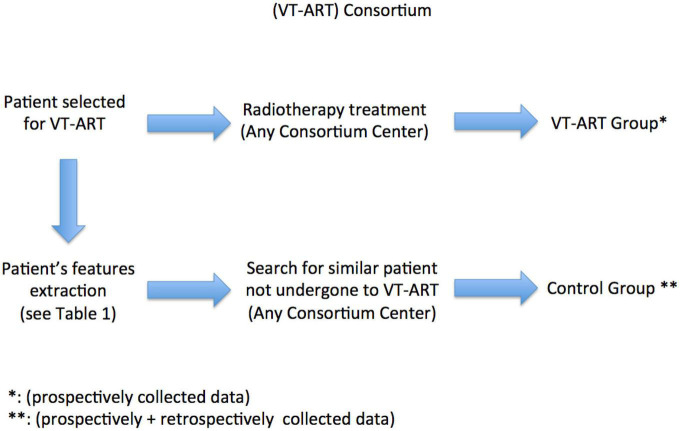
Overview of the VT-ART study.

Figure 1 depicts the design of the VT-ART study.

## Radiotherapy

Ventricular tachycardia ablation through radiation therapy will be administered through image-guided radiotherapy (IGRT) by a linear accelerator (Linac) (e.g., TrueBeam–Varian Medical Systems, Palo Alto, CA), an SBRT-dedicated Linac, including a dedicated Linac with gating delivery (e.g., TrueBeam Edge Linac, Varian Medical Systems, Palo Alto, CA) with 6 MV flattening filter-free photons and dose calculation algorithm Acuros (Eclipse Version 15.6.04 Varian Medical Systems, Palo Alto, CA), or a MR-guided Linac (i.e., ViewRay MRIdian). Selection of a specific Linac type for each patient will be addressed based on the best personalization achievable, after a multidisciplinary discussion of the patient-specific clinical background, needs, compliance, and technical possibilities. The target area (TA), in terms of the clinical target volume (CTV), set-up margin (SM), and internal target volume (ITV), will be determined for each patient on a 3D computer tomography reconstruction based on CT scan or MRI (not performed if the patient has no MRI-conditional ICD), 12-lead ECG data, and electroanatomical mapping (EAM).

Three-dimensional EAM will be performed by three different systems on the basis of availability of the three workstations [Carto 3 system, Biosense Webster Inc. (Diamond Bar, CA, USA), Ensite Precision Cardiac Mapping Navx, Abbott (North Plymouth, MN, USA), and RHYTHMIA HDx™ Mapping System, Boston Scientific, Copyright IBM Corporation (Marlborough, MA, USA)]. Electrical information from the EAM and information from imaging will be combined to build a volumetric target for VT-ART (2).

Electroanatomical mapping data will be fused with CT-reconstructed 3D models applying the previously described approach for non-invasive EAM, through a series of electrode strips (worn by the patient) containing 256 electrodes (BioSemi, Netherlands), with small radiopaque markers attached at the location of the electrodes, to assist with visualization on cardiac imaging. A gated chest CT scan with 3 mm axial resolution was obtained to provide patient-specific heart-torso geometry and the location of the body surface electrodes relative to the heart (1–3). Since there is still no unique, standardized approach for TA delineation for those clinical scenarios, after the anatomical definition of the cardiac areas to be irradiated by EAM, an indirect TA definition by manual delineation of the EAM on a four-dimensional gated simulation CT scan, independently double-checked by two different radiation oncologists and cardiologists referring to the same EAM, will also be allowed.

A 2–5-mm margin will be provided to the TA to determine the planning target volume (PTV). The prescribed dose will be 25 Gy in a single fraction to 80% isodose. A 4D-CT will be applied to account for respiratory motion and either cone-beam computed tomography (CBCT)-based or MR-guided Linac positioning with the addition of either breath hold (BH) or free-breathing respiratory gating, depending on the patient’s compliance. If BH could be applied, deep inspiration breath hold (DIBH) should be preferred, but mid inspiration breath hold (MIBH), mid expiration breath hold (MEBH), and deep expiration breath hold (DEBH) are allowed if more suitable for planning.

Volumetric modulated arc therapy (VMAT) will be applied by multiple partial arcs (PA) depending on the arrhythmogenic scar volume and morphology to cover the TA. If an MR-guided Linac is used, static intensity-modulated radiotherapy (IMRT) will be applied.

Dose constraints for organs-at-risks and dose to target will follow the indications from the AAPM report Task Group 101 for single-fraction SBRT (4).

Our trial will combine two IGRT procedures to increase safety: intra-fractional and inter-fractional motion management devices and procedures are mandatory to be used. Volumetric imaging (CBCT- or MR-guided) scans before delivering each PA with regard to inter-fractional monitoring. Intra-fraction monitoring through the gating system is also mandatory. Continuous intra-fraction monitoring through an optical surface monitor system (OSMS) will be applied if available (e.g., TrueBeam Edge Linac, Varian Medical Systems, Palo Alto, CA, USA). Online adaptive procedures will be applied for the MR-guided Linac.

## Outcome measures

The primary outcome is investigating the difference in overall cardiovascular survival among the group undergoing VT-ART and the one not exposed to the innovative procedure.

The primary efficacy endpoint is a statistically significant difference in overall cardiovascular survival between the group undergoing VT-ART and the one not exposed to the innovative procedure; this will be defined in months and calculated from the time of the last procedure (represented by the last RFA for the control group and VT-ART for the innovative one, respectively).

The secondary outcome is evaluating the difference in ventricular event-free (referred to VT/VF, appropriate shock or ATP as recorded by the ICD) survival after the last procedure between the two groups (i.e., the single or last RFA in the control group and VT-ART in the innovative group). The respective endpoint is a statistically significant difference in ventricular event-free survival (in months) after the last procedure between the two groups.

An additional secondary aim is to evaluate the reduction in the number of VT episodes compared to the pre-treatment period between the two dynamic cohorts that will be investigated.

Ventricular tachycardia recurrence will be defined as evidence of monomorphic VT at ICD interrogation (either self-limiting or terminated by appropriate shock therapy or ATP). The number of VT episodes, defined as VT burden, in the 3 months before and after the procedure will then be compared.

The respective secondary endpoint will be evaluated facing 3 months before the last procedure (i.e., either RFA or VT-ART) and 3 months after.

We did set a blanking period after VT-ART before evaluate the effect on VT burden; in a recent study by Kautzner et al., the authors supported the pre-clinical theorem of myocardial apoptosis (up to 3 months post-stereotactic ablation) followed by a creation of fibrotic lesion (6–9 months post-stereotactic ablation) in the irradiated region (5). Consequently, we propose a 3-month-blanking period after the procedure.

Since a blanking period of 3 months after the procedure will be applied, the secondary endpoint will evaluate the number of VT episodes interval accounted for the last 3 months before the procedure and of the 3–6 months after it.

Other secondary objectives include identifying the benefits of VT-ART on cardiac function, as evaluated through an electrocardiogram, echocardiographic, biochemical variables, and patient’s quality of life.

In particular, we will evaluate the reduction or suspension of antiarrhythmic drug use compared to the baseline and the improvement of cardiac parameters (such as LVEF, left ventricular end-diastolic volume/diameter, ventricular strain, end-systolic volume/diameter, RVEF, and TAPSE for right ventricular function).

Analyzed biochemical variables will include heart failure biomarkers, such as NT-proB-type natriuretic peptide (NTproBNP), troponin (hs-TnI), and inflammation markers, such as polymerase chain reaction (PCR), tumor necrosis factor-alpha (TNF-alpha), and interleukin 6 (IL-6).

Improvement in the patient’s quality of life will be assessed compared to the pre-treatment period using the SF36 scale.

The patient’s QoL will be assessed before and after VT-ART using the SF36 scale (6); the post-treatment score will be compared to the pre-treatment period’s one to assess potential improvement. The post-treatment QoL evaluation will be performed at 3, 6, and 12 months after VT-ART. Once centers join the consortium, the SF36 QoL scale will be offered (although not mandatory) to all patients undergoing RFA: Before and at 6 months after conventional therapy.

Secondary outcome analyses will investigate the improvement of QoL scores after VT-ART and how QoL scores change the comparison between VT-ART and RFA.

Further secondary outcome analyses will be developed through imaging: Contrast infusion MRI and CT scan will be performed as required but not mandatory (if not contraindicated due to ICD) in the experimental arm.

### Statistical considerations and sample size for matched pair analysis

A descriptive analysis will summarize the total number and stratification of patients with RFA and VT-ART.

We will perform a comparative analysis between RFA and VT-ART in patients with structural heart disease and refractory VT. Each patient who will be treated by VT-ART will be compared with two patients in the control group treated with RFA and best standard care but without VT-ART, on the basis of the following matching factors: Etiology of cardiomiopathy (ICM, NICM, and ACM), ventricular arrhythmia presentations (sustained ventricular tachycardia; incessant sustained ventricular tachycardia; and electrical storm), diagnostic tools used for scar definition (CT, MR, EAM, and ECG), arrhythmogenic focus anatomical intracardiac location (on the basis of VE exit by ECG and by activation map of VT), TA volume (endocardial electroanatomic substrate mapping area/volume in all cases; and epicardial mapping area/volume in case of 3D epicardial electroanatomic substrate mapping), heart volume, heart-to-TA volume, rate number of previous RFA attempts, type of previous RFA attempts (endocardial and epicardial), time (in days) between the first RFA attempt and VT-ART, time (in days) between the last RFA attempt and VT-ART, previous percutaneous stellate ganglion blockade, time (in days) between the percutaneous stellate ganglion blockade and VT-ART previous use of amiodaron, NYHA group, LVEF, BMI, age, and gender.

As mentioned, applied matching factors are summarized in Table 2.

Continuous data will be expressed as mean ± standard deviation, as appropriate, for all variables collected from the entire population or specific subgroups. A comparative analysis for subsequent levels of qualitative variables through the chi-square test and quantitative variables (scores on quality of life) with the *T*-test will be performed.

Non-parametric tests will be provided where the population distribution does not have a known form (normal Gaussian).

The differences in baseline and follow-ups between the same group will be analyzed with repeated measures approach: using the Friedman test and MANOVA for continuous variables and McNemar or Cochran’s *Q*-tests for categorical ones.

The significance of the tests is fixed with *p* < 0.05.

#### Sample size

The calculation of the sample size considered followed assumptions:

- A total of 95% confidence level;
- The drop-out rate of 10%;
- Expected difference between the two treatments for 3-month and 6-month VT burden in terms of overall response rates: 20% higher in arm including VT-ART compared to the control group;
- Power of 80%.

To improve clinically adequate balancing, we will enroll two matching paired patients in the group not undergoing VT-ART for each patient who undergoes VT-ART in a 2:1 ratio, favoring a larger proportion of patients not undergoing the innovative procedure (i.e., VT-ART).

Under these hypotheses, to detect a 20% improvement in the VT burden control rate in the VT-ART group vs. the control group, we calculated the sample size (in a 2:1 ratio) upon enrolling 149 patients: 100 in the non-exposed control group and 49 in the VT-ART group. Progressively, on a multicentric basis supervised by the promoting center in the VT-ART consortium, for each VT-ART patient enrollment, a matched pair patient profile according to the predefined features will be shared with the consortium to enroll two similar patients who have not undergone VT-ART. For enrollment into the control group, recruitment of clinical cases from retrospective case series within the multicentric consortium will also be allowed, with the only condition to be not earlier than 5 years from the enrollment of the matched pair patient in the VT-ART group (to avoid the possible influence of a too early approach for standard procedures).

All statistical analyses will be performed using SPSS^®^ version 25.0 software (©Copyright IBM Corporation 1994, 2017).

Due to the potential difficulty of enrolling the patients through the complete match of all matching factors, a protocol rule will be applied through patient’s collection.

Once the planned number of the innovative procedure will be collected, the trial will put a 6-month observation time. If after that, 60% of the planned conventional arm patients will be collected (by all matching factors and in a 2:1 ratio), a further 3 months will be provided to reach the planned accrual. If that would have not been the case, then the patient’s enrollment will be allowed by mandatory matching factors only (only beyond that point). The detail of “mandatory matching factors” is reported in Table 2. The final analysis will be performed by the whole group and subgroup analyses, discriminating patients collected through “all matching factors” and by “mandatory-only.”

The final analysis will not be performed at all if less than a 1:1 ratio (conventional: Innovative group) of patient’s enrollment will be collected, and the study will be closed.

The final analysis will be performed specifying that the planned accrual is not met if at least a 1:1 ratio (conventional: Innovative group) enrollment will be accomplished; results will be explored and cautiously evaluated.

### Toxicity evaluation

Radiotherapy-related toxicity will be defined using the Common Terminology Criteria for Adverse Events (CTCAE) v5.0. All adverse events, regardless of the toxicity grade and all-cause mortality, will be reported in the final study. Patients will be re-evaluated at 2 years of follow-up for late-onset toxic effects.

The secondary safety endpoints for VT-ART include the evaluation of all adverse events, which are classified as follows:

• Acute, during the patient’s hospital stay;
• Subacute, in the first 90 days from discharge;
• Chronic, 6–12 months following discharge.

### Follow-up

Follow-up will be conducted for 36 months after treatment administration.

Patients prospectively enrolled within the consortium and treated by VT-ART will undergo ICD interrogations for up to 36 months after enrollment.

After a blanking period of 3 months, regular ICD interrogations [with intracardiac electrogram (EGM) storage of VT/VF or appropriate shock or ATP] will be prospectively collected for patients treated by VT-ART at 6, 9, 12, 18, 24, 30, and 36 months after the VT-ART procedure.

The ICD interrogation will collect the events along the last 3 months ahead of each follow-up evaluation.

Patients prospectively enrolled within the consortium and treated by RFA will undergo the same follow-up schedule if possible according to the clinical patient’s need and compliance.

Patient data retrospectively collected within the consortium and treated by either RFA or VT-ART will be retrospectively extracted at the same time points of prospective follow-up.

The final analysis will be performed only for patients with available data for the mentioned time points, either retrospectively or prospectively collected.

Quality of life will be assessed before the procedure (for prospectively enrolled patients candidate to VT-ART and RFA) and at 3, 6, and 12 months after therapy. Improvement in the patient’s quality of life will be assessed compared to the pre-treatment period using the SF36 scale.

Patient follow-up will thus, briefly, include:

• ICD interrogation (with EGM storage of VT/VF) at 6, 9, 12, 18, 24, 30, and 36 months from treatment.
• A total of 12-lead ECG at 3, 6, and 12 months.
• Echocardiography at 3, 6, and 12 months.
• QoL assessment using the SF36 scale (7) will be performed at 3, 6, and 12 months for patients undergoing VT-ART.
• QoL assessment using the SF36 scale will be performed at 6 months for patients undergoing RFA.

### Registration

The trial is promoted by Fondazione Policlinico A. Gemelli IRCCS, Rome (Italy). The clinical and investigational procedures will be based on approval from the local ethics committee. Each interested center will submit the protocol to its ethics committee for approval before accrual. After approval, the center will receive a dedicated electronic case report form (CRF). Eligible participants who provide consent and meet the inclusion criteria are anonymously registered in the CRF by assigning a numerical code. Final stratification of the enrolled patients will be performed globally to enhance the homogeneity and balance of the final dataset.

### Ethics considerations

The trial will be conducted in compliance with the approved protocol, Declaration of Helsinki 2008, principles of Good Clinical Practice (GCP), and Italian National Normative for clinical experimentation. Upon signing the protocol, every investigator will provide consent for the procedure and instructions in the protocol and run the study according to the GCP, Declaration of Helsinki, and National Normative. Every amendment to the study will be registered and submitted to the ethics committee. Our trial protocol and its attached material have been approved by the Ethics Commission of Fondazione Policlinico Gemelli IRCCS (Rome, Italy). Every participant center must submit our protocol to their respective ethics commission before enrolling patients.

## Discussion and final considerations

Sustained monomorphic VT is a potentially life-threatening condition that often affects patients with structural heart diseases. VT recurrence poses a severe threat to health and quality of life. Long-term management of recurring VT relies mainly on ICDs, pharmacological control (i.e., class III antiarrhythmic), and RFA of the pathological arrhythmogenic substrate.

New long-term solutions are currently being sought for patients in whom VT is refractory to conventional treatment strategies or in whom it cannot be performed. In patients with structural heart disease, the rate of VT recurrence after RFA has been reported to be between 25 and 50% (8). Intrinsic technical aspects of the procedure (e.g., difficult anatomical location of the arrhythmic substrate) as well as patient-related factors (e.g., patient frailty) may limit RFA feasibility in a portion of the population. Patients with structural heart disease are often fragile and have a varying number of comorbidities. Therefore, procedure invasiveness could be a limiting factor in the choice of the best treatment strategy (9).

Cuculich et al. first investigated the efficacy and safety of stereotactic radiation for the ablation of VT in a five-patient case series, which was later followed by the publication of a phase I/II prospective study by the same authors, including 19 patients (1, 2). Both studies reported a statistically significant reduction (99.9 and 94%, respectively) in VT episodes after a blanking period of 4 weeks. Subacute and chronic treatment-related side effects ranged from asymptomatic pericardial effusion to radiation-induced pericarditis and pneumonitis and were successfully treated with corticosteroids. Since then, a growing number of scientific articles have been published on this matter.

Overall, VT-ART has been proven to be an effective and adequately safe intervention in other studies (10, 11). Its non-invasive nature makes it a safe alternative for patients who are not suitable for percutaneous RFA (9). Nevertheless, despite the promising results detected by sporadic reports, limited case series, and systematic reviews (12), we still do not know if and at what level VT-ART (and STAR in general) could significantly and effectively improve the clinical scenario of such complex malignancies.

## Conclusion

Our trial will provide insight into the efficacy and safety of VT-ART through a matched pair analysis, by an observational, multicentric study (with prospective and retrospective data collection) *via* subgroup stratification into dynamic cohorts of two groups of patients with or without VT-ART in a multicentric consortium.

## Ethics statement

This study was reviewed and approved by the Ethics Commission of Fondazione Policlinico Gemelli IRCCS (Rome, Italy). Written informed consent will be obtained from all participants for their participation in this study and every participant center must submit our protocol to their respective ethics commission before enrolling patients.

## Author contributions

All authors listed have made a substantial, direct, and intellectual contribution to the work, and approved it for publication.
